# Treatment of shoulder ulcers in sows – rubber mats and zinc ointment compared to chlortetracycline spray

**DOI:** 10.1186/1751-0147-55-12

**Published:** 2013-02-15

**Authors:** Marianne Kaiser, Charlotte S Kristensen, Poul Bækbo, Lis Alban

**Affiliations:** 1 Department of Veterinary Research and Development, Pig Research Centre, Danish Agriculture & Food Council, Vinkelvej 11 – 13, P.O. Box 50, DK-8620 Kjellerup, Denmark; 2 Danish Agriculture & Food Council, Department for Food Safety and Veterinary Issues, Agro Food Park 13, DK-8200, Aarhus N, Denmark

**Keywords:** Shoulder ulcer, Wound, Pressure sore, Rubber mat, Sow

## Abstract

**Background:**

Shoulder ulcers can have a significant impact on the welfare of sows. In Denmark, rubber mats are used for treatment of shoulder ulcers. The objective of the study was to compare a treatment consisting of a combination of rubber mats and zinc ointment with the effect of local antibiotic spray (chlortetracycline) on shoulder ulcers.

**Methods:**

A total of 304 sows with shoulder ulcers were observed shortly after farrowing (day 1) and on days 14 and 21 after the first observation. The sows were paired according to the grade of the shoulder ulcer using the official Danish scoring system. From each pair of sows, one sow was allocated to mat group (M) and the other to an antibiotic group (A) in a random way. In the M group, rubber mats were placed on the floor, and the ulcers were treated with zinc ointment once a day. In the A group, sows were treated with antibiotic spray daily. The size of the shoulder ulcer was measured manually on a continuous scale on days 1, 14 and 21. The data were analysed by use of two multivariable models where the response was the area of the shoulder ulcer on day 14 and day 21, respectively. Explanatory variables were treatment, herd, parity, body condition and size of ulcer on day 1. If a sow had an ulcer on both shoulders, the shoulder with the largest ulcer was selected.

**Results:**

The treatment consisting of mats and zinc ointment had a statistical significant effect on the size of the shoulder ulcer on day 14 and day 21 compared to daily treatment with antibiotic spray. For lean sows that were kept on rubber mats and zinc ointment, the average shoulder ulcer was significantly smaller on day 14 (3.8 cm^2^ versus 9.5 cm^2^ when antibiotic spray was used) and day 21 (3.4 cm^2^ versus 6.6 cm^2^) compared to lean sows that were only treated with antibiotic spray. For fat sows this was also the case when using the lesion on day 21 as the response (2.0 cm^2^ versus 5.7 cm^2^) but not on day 14. Moreover, the size of the lesion on day 1 was statistically associated with the size of the lesion on day 14 and on day 21. The treatment was equally effective in the three herds.

**Conclusions:**

Rubber mats and daily smearing with zinc ointment slow progression and contribute to the healing of shoulder ulcers compared with housing on slats and daily spraying with antibiotics. It is recommended to place a rubber mat in the farrowing crate at the first sign of shoulder ulcers.

## Background

Shoulder ulcers might cause pain [1] and have a negative impact on the welfare of sows. It is impossible to estimate the exact loss of production, but in Denmark affected sows are being euthanized for this reason. In 2008, a prevalence of 17.2% was found in 98 Danish herds [2]. Therefore, shoulder ulcers have received much attention in Denmark.

Generally, lactating sows are housed on partial or fully slatted floors in confined crates during the whole nursing period. In Denmark straw is allocated for keeping sows occupied and nesting but not for bedding. Shoulder ulcers are primarily observed in the farrowing unit [3,4] and poor body condition, high parity, scars from previous shoulder ulcers, lameness and breed are known risk factors for the disorder [5].

Shoulder ulcers are caused by prolonged pressure on the scapular spinal process, resulting in ischemia and subsequent necrosis [6,7]. Decompression and correct positioning are important parts of therapy for human patients with pressure sores [8]. Even though experience from human medicine cannot be directly applied to sows, rubber mats have been recommended as therapy together with topical treatment with various ointments. There is only limited documentation of the effect of these treatments. A single Canadian study has shown that shoulder ulcers healed faster if sows were housed in farrowing crates with a rubber mat on the floor compared with sows housed in farrowing crates with fully slatted or solid steel floors [9].

The objective of the study was to compare a combination of rubber mats and zinc ointment (25% zinc oxide) with local antibiotic treatment (chlortetracycline spray) on healing of shoulder ulcers in sows. Rubber mats and zinc ointment is a treatment commonly used by Danish farmers.

## Methods

### Herds

Three sow herds with a history of recurrent problems with shoulder ulcers were included in the study. The herds had a closed production and were high-health herds, included in the Danish Specific Pathogen Free system (http://www.spf-sus.dk/sus/en-GB/ - link visited January 23, 2013). They had fully slatted plastic floors in the farrowing pens, measuring 1.6 × 2.5 m^2^. The sows were housed in confined crates from 1 week before farrowing and the whole nursing period (3 weeks). In herd 1 and herd 2, the sows were fed liquid feed. In herd 3, they were fed dry feed. All sows were of the Danish cross-breed (Landrace/Yorkshire).

### Classification of shoulder ulcers

In Denmark, shoulder ulcers have been classified clinically using an officially accepted pathoanatomical scale since 2003 [10,11]. Although this scale was not developed for clinical use, it has been the most efficient means of obtaining a clinical evaluation until now. It classifies the ulcers into five different grades ranging from 0 to 4. Grade 0 indicates no ulceration. In grade 1 the ulceration involves only the epidermal layer of the skin. In grade 2 the epidermal layer and dermal layer are affected. In grade 3 the subcutaneous layer is ulcerated, and, finally, in grade 4 the bone (*tuber spina scapula*) is exposed. In accordance with this system, sows were not recorded as having a shoulder ulcer if the skin above the shoulder was only hyperemic.

### Sample size

The number of sows to be included in the study was estimated to be 300. The calculations were based on the following sample size considerations. The primary variable was the proportion of sows in which the ulcers had decreased in size on day 14 after farrowing. It was expected that the size of the ulcers would decrease among 50% of sows that were kept on rubber mats compared with 10% among sows that received antibiotic spray treatment instead. The estimate was based on a power of 80% and a significance level of 1%.

### Selection of sows and allocation to treatment

A total of 304 sows were observed from September 2005 to September 2006 (93 in herd 1, 107 in herd 2, and 104 in herd 3). All sows were observed three times. At the first observation (day 1), sows with either grade 1 or grade 2 ulcers were identified visually and included in the study. No sows with more severe shoulder ulcers (grades 3 or 4) were found on day 1. If sows developed grade 3 or 4 during the study, they were euthanised due to animal welfare considerations. Since the focus of this study was to investigate the effect of rubber mats on sows with shoulder ulcers, sows without ulcers were not included. The sows were paired according to grade of ulcer at day 1. If a sow had lesions on both shoulders, the largest ulcer was used for pairing. If there was an extra sow which was not possible to pair, she would still be included in the study. After paring, the sows were randomly divided into either group M (mats and zinc ointment) or group A (antibiotic spray). The treatment in group M consisted of placing a rubber mat in the pen and smearing the shoulder ulcers once a day with a sufficient amount of zinc ointment to cover the lesion. The mat chosen for the study (Atlas 18 mm, Kraiburg Elastik GmbH, Tittmoning, Germany) measured 70 cm × 110 cm × 1.8 cm. The degree of rubber hardness was Shore A 60 ± 5. This mat did not cover the total area of the sow’s body, but would always protect the shoulder from exposure to the floor. The zinc ointment was Apotekets Baby Zinksalve, 100 ml. 25% Zinc oxide, no. 208530 (Apotekernes A.m.b.a, Skovlunde, Denmark). An additional documentation file shows this in more detail [see Additional file 1]. Sows in group A were sprayed one second with an antibiotic spray (chlortetracycline HCL, 2.45%, Cyclo Spray Vet, Eurovet Animal Health B.V. Bladel, the Netherlands) once daily, because it was considered unethical if this group of sows did not receive any treatment. The study was not blinded, because it was obvious for the observers to spot the difference between the two groups.

### Recordings of size of shoulder ulcer and explanatory variables

Sows farrowed continuously within the herds. On day 1, observations were made on sows that had farrowed within the last week, mostly 3 – 5 days before the visit. The area of the shoulder ulcer was estimated by drawing the contour of the ulcer on a transparency sheet (Esselte, Dataline, code 57167). The area of the sheet was subsequently cut out, by the same person using the same scissors, and then weighed. The weight of the sheet was converted into surface area. This procedure was repeated on day 14 and 21 after the first observation.

It has been shown that shoulder ulcers occur more commonly on the right side than on the left side [4]. Thus, the size of a shoulder ulcer might also depend on which side it is located. Therefore, the location of the shoulder ulcer (left side/right side) was recorded. The parity of the sow was noted and the body condition score was estimated at all observations using a scale developed by the Danish Pig Research Centre, Danish Agriculture & Food Council [12]. In this scale the sows are classified into four classes, with class 1 consisting of lean sows, class 2 of thin sows, class 3 of medium sows, and class 4 of fat sows. For practical reasons the observations of the ulcers were made by two persons. It is acceptable for three reasons. The first observer trained the second observed to reduce variation between observers. The drawing of ulcer size is considered a fairly precise tool, which reduced the inter-observer variation and finally group M and A were assessed by the same person at every visit on a farm.

The average size of the shoulder ulcer was calculated for group M and A, for each of the three visits, respectively.

### Statistical analysis

Parity was dichotomized into parity >7 (old) and parity ≤7 (young). Body condition score was dichotomized too, so lean sows had score 1 or 2 and fat sows had score 3 or 4. The dichotomizations were based on a prior study of the effect of various risk factors on the risk of developing shoulder ulcers [13].

The size of the ulcer on days 14 and 21 of the individual sows respectively, was used as a response in each of their linear mixed models (PROC MIXED in SAS) [14]. If a sow had two ulcers, the shoulder with the largest ulcer was chosen. The potential explanatory variables were treatment (M/A), body condition (lean/fat), parity (young/old), herd (1, 2, and 3), side of the ulcer (left/right), and size of the ulcer on day 1. The grade of the ulcer was not included as a risk factor because it was strongly correlated with the size of the ulcer on day 1 (data not shown). All risk factors were screened for association with the each of the two response variables. The side of the ulcer (left/right) was left out from the subsequent analyses because initial analyses showed a lack of significant association with the response variables. To ensure that the allocation of sows into treatment groups was performed at random, a model was made which included the size of the ulcer on day 1 as a response.

All factors that were significant at 5% level in the univariable analysis were offered to a multivariable model. Both forward and backward selections were used to find the most appropriate model. Plausible interaction terms were tested for statistical significance. Because it was known that there might be an impact of body condition on the effect of treatment [15], a re-categorisation was made by combining the two variables body condition and treatment into a new variable with four levels. This variable was offered to the models where preliminary analyses had showed a statistical significant interaction term between body condition and treatment. All tests were performed as two-sided tests. The models were validated by inspection of residuals and by running the models with and without outliers and comparing the model estimates. An inspection of the residuals and the original data showed that the few outlying observations did not have a strong impact on the final results and were therefore kept in the analyses. Least square means were calculated for the class variables that were included in the final model, and the degree of statistical difference between the different levels of these variables was estimated.

## Results

### Descriptive statistics

**Figure 1:**
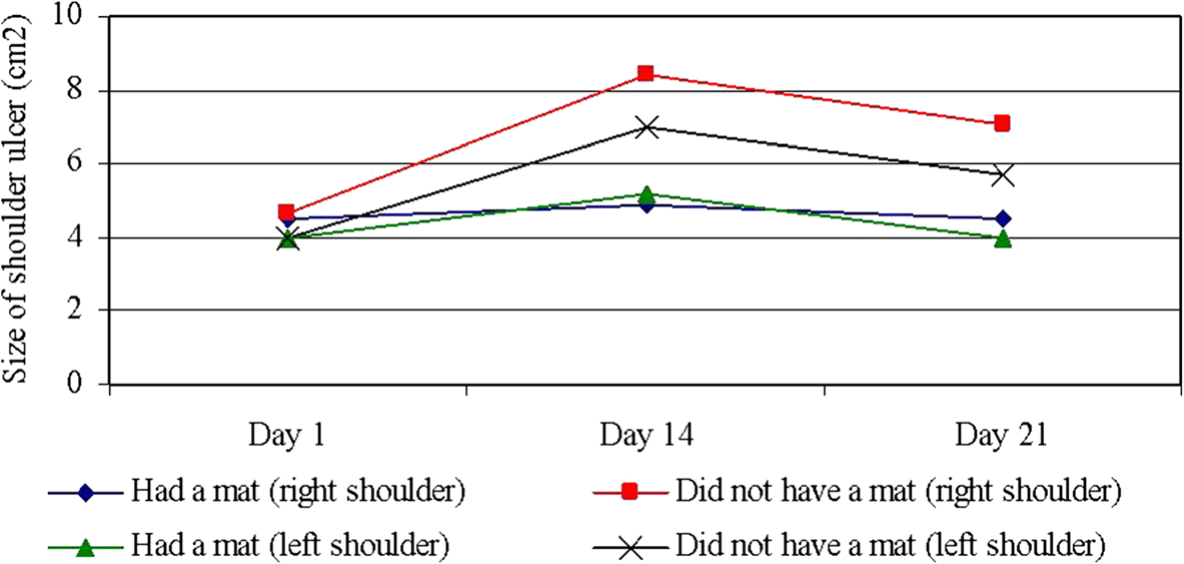
**Descriptive graphical presentation of average size of shoulder ulcers in two groups of 304 sows with shoulder ulcers that either received a rubber mat and zinc ointment or cutaneous antibiotic spray.** The visit on day 1 was conducted during the first week after farrowing. The following visits were conducted 14 days and 21 days after the first visit.

Table 1 describes how the four variables - parity, body condition, side of ulcer, and herd - were distributed according to the type of treatment (mat and zinc ointment versus antibiotic spray) on day 1. For the entire study period, the prevalence of the shoulder ulcers was 14% in herd 1, 17% in herd 2 and 7% in herd 3. During this period the number of sows included in the study was reduced from 304 to 226. The 78 sows leaving the study were either weaned (44), slaughtered (3) or transferred to a hospital pen/euthanised because of various health problems (30 sows). A larger number of lean sows from group A (34 sows) left the study population compared to group M (23 sows). It is unknown why this occurred. The median size of the shoulder ulcers was 1.7 cm^2^ on day 1. On day 14, the ulcers had generally increased in size. For the sows which received a rubber mat and zinc ointment the median size was 2.0 cm^2^ versus 7.8 cm^2^ for sows that only received cutaneous antibiotic treatment (Table 2). Likewise, on day 21, the shoulder ulcers were slightly smaller: 1.3 cm^2^ for sows with mats and zinc ointment versus 5.1 cm^2^ for the other sows (Table 2). Hence, there was one development for the sows offered a rubber mat and another development for sows, which were not offered a rubber mat (Figure 1).

**Table 1:** Explanatory variables* among 304 Danish sows with shoulder ulcers

**Variable**	**Levels**	**Mats (M) Number (%)**	**Antibiotic (A) Number (%)**	Total**
Parity	Young (1–6)	138 (60.4)	136 (49.6)	
	Old (7–14)	15 (50.0)	15 (50.0)	304
Body condition	Lean	98 (53.3)	86 (46.7)	
	Fat	54 (46.2)	63 (53.9)	301
Side	Left	38 (41.8)	53 (58.2)	
	Right	113 (54.3)	95 (45.7)	299
Herd	1	47 (50.5)	46 (49.5)	
	2	53 (49.5)	54 (50.5)	
	3	53 (51.0)	51 (49.0)	304

*: Parity, body condition, side of ulcer, and herd – divided according to type of treatment (M: mat and zinc ointment versus A: cutaneous antibiotic treatment alone).

**: There were two or three missing observations for two of the explanatory variables.

**Table 2:** Size of ulcers on day 1, day 14 and day 21 among 299 sows treated for shoulder ulcers

**Day of observation**	**Number of observations**	**Median size of ulcer (cm**^**2**^**)**	**90% Percentile interval (cm**^**2**^**)**
Day 1^a^ – based on day 14			
Mat and zinc	151	1.8	0.0-10.7
Cutaneous antibiotic treatment	148	1.6	0.0-12.0
Day 1^b^ – based on day 21			
Mat and zinc	117	1.4	0.0-11.0
Cutaneous antibiotic treatment	103	1.0	0.0-9.8
Day 14			
Mat and zinc	151	2.0	0.0-14.2
Cutaneous antibiotic treatment	148	7.8	0.0-19.4
Day 21			
Mat and zinc	117	1.3	0.0-10.5
Cutaneous antibiotic treatment	103	5.1	0.0-17.1

^a^: This reflects the side of the shoulder where the ulcer was largest on day 14, in case there was an ulcer on both shoulders.

^b^: This reflects the side of the ulcer that was largest on day 21.

### Multivariable analyses

In the following, the results of the multivariable models are presented. First, the results of using ulcer size on day 1 as the response were presented. The aim of this model was to study whether the allocation into treatment groups had worked satisfyingly. Next, the results of day 14 and day 21 are presented.

### Ulcer size on day 1

Only the variable herd was significantly associated with ulcer size on day 1 (Table 3). This implies that the size of the ulcer on day 1 did not differ between the two treatment groups nor was there an association with body condition or parity (Table 3). Hence, the randomisation process regarding allocation of sows into the two treatment group performed well.

**Table 3:** **Explanatory variables in three different models studying treatment of shoulder ulcer in sows**^**a**^

**Day of observation**	**Number of observations included**	**Model**	***P*-value for statistical association between explanatory variable and response**						
			**Treatment**	**Body condition**	Treatment * Body condition	**Parity**	**Size of ulcer on Day 1**	**Herd**	Herd * treatment * Body condition
1	299	Full model	0.72	0.65	0.67	0.86	Not relevant	0.0060	Not relevant
		Final model with significant variables only	NS	NS	NS	NS	Not relevant	0.0047	Not relevant
14	299	Full model	<0.0001	0.30	0.0004	0.09	<0.0001	0.0014	0.14
		Final model with significant variables only	<0.0001	0.10	0.0005	NS	<0.0001	0.0038	NS
14^b^	299	Final model with significant variables only^c^	<0.0001			NS	<0.0001	0.0038	NS
21	220	Full model	<0.0001	0.29	0.45	0.05	<0.0001	0.23	0.10
		Final model with significant variables only	<0.0001	NS	NS	0.03	<0.0001	NS	NS
21^b^	220	Final model with significant variables only^c^	<0.0001			NS	<0.0001	NS	NS

^a^: For each sow at each time of observation the size of the largest ulcer was chosen. NS: Non-significant implies that the *P*-value was >0.05.

^b^: The side of lesion on day 1 was identified separately for the model describing lesion sizes at day 14 and day 21, separately.

^c^: In this model a new variable was created based on a combination of the two variables Body condition (lean/fat) and Treatment (rubber mats and zinc ointment versus cutaneous antibiotics). This new variable had four levels.

### Ulcer size on day 14

The following variables were significantly associated with the size of the ulcer on day 14: size of ulcer on day 1, treatment, an interaction term between treatment and body condition, and herd. Although body condition was not significant (*P*=0.10), the two variables body condition and treatment were subsequently combined into a new variable with four levels. This new variable was also offered to the model instead of the two main effects and their interaction term (Table 3). The parameter estimates and associated *P*-values of this model are presented in Table 4. The interaction term between herd, treatment and body condition was non-significant (*P*=0.14); hence, herd had no impact on the effect of treatment on the size of the ulcer on day 14. The least square means showed that the effect of treatment was statistically significant for lean sows (with mat: size of ulcer on day 14 was 3.8 cm^2^ versus antibiotic spray 9.5 cm^2^ – *P*-value for difference <0.0001 – Table 4). For fat sows, the effect was smaller and not significant (with mat: 4.9 cm^2^ versus antibiotic spray 6.3 cm^2^- *P*-value for difference 0.15 (Table 4). The parity of the sow (≤7 or >7) did not impact statistically significant on the size of the lesion on day 14 (*P*=0.09 – Table 3). Neither was there an effect of parity on the effect of treatment (interaction tem between parity and treatment *P*=0.67 – Table 3). The least square means for herd showed that the size of the ulcer were statistically significant higher in herd A compared to herd B and C (7.6 cm^2^ versus 5.5 cm^2^ and 5.3 cm^2^- Table 4).

**Table 4:** Results of two different multivariable models describing effect of treatment of shoulder ulcers on 299 Danish sows

**Model describing**	**Explanatory variables / levels**	**Parameter estimate (cm**^**2**^**)**	**S.E. (cm**^**2**^**)**	***P*-value of variable**	***P*-value of levels of class variable**	Least square means* (cm^**2**^**)**
Size of ulcer on day 14	**Size of ulcer on day 1**	0.52	0.41	<0.0001		
	Body condition *			<0.0001		
	**Treatment**	−2.50	0.83		0.0027	3.78^a^
	Lean sow + rubber mat	3.15	0.85		0.0003	9.45^b^
		−1.36	0.94		0.1512	4.92^ad^
	Lean sow + cutaneous antibiotics	0.00	*		*	6.28^cd^
	Fat sow + rubber mat			0.0038		
	Fat sow + cutaneous antibiotics	2.28	0.74		0.0023	7.56^a^
		0.19	0.71		0.7836	5.47^b^
	**Herd**	0.00	*		*	5.28^b^
	Herd 1					
	Herd 2					
	Herd 3					
Size of ulcer on Day 21	**Size of ulcer on day 1**	0.51	0.41	<0.0001		
	Body condition * Treatment			.0001		
		−2,32	0.80		0.0043	3.40^a^
	Lean sow + rubber mat	0.82	0.87		0.3447	6.55^bd^
		−3.71	0.91		<0.0001	2.04^ac^
	Lean sow + cutaneous antibiotics	0.00	*		*	5.73^d^
	Fat sow + rubber mat					
	Fat sow + cutaneous antibiotics					

*: The least square estimates were based on the final model and hence take into account the effect of the size of the ulcer on day 1.

Different superscripts indicate that the least square estimates differed statistically significant between two groups of sows.

### Ulcer size on day 21

The following variables were significantly associated with the size of the ulcer on day 21: size of ulcer on day 1 and the variable that combined the variables treatment and body condition (Table 3). This model was preferred to a model that included parity, treatment and size of lesion on day 1, although parity had a *P*-value of 0.03 (Table 3) because of a strong correlation between body condition and parity (*P*=0.0036). There was no effect of herd on the size of the ulcer on day 21. In line, the interaction term between herd, treatment and body condition was non-significant (*P*=0.10 – Table 3); hence, herd had no impact on the effect of treatment on the size of the lesion on day 21. The least square mean results showed that the effect of treatment was statistical significant both for lean and fat sows. For lean sows with mats, the ulcer on day 21 was 3.4 cm^2^ versus 6.6 cm^2^ for lean sows which received antibiotic spray (*P*-value for difference =0.0001). For fat sows, the effect was slightly larger (with mat: 2.0 cm^2^ versus 5.7 cm^2^- *P*-value for difference <0.001 (Table 4). There was no effect of parity on the effect of treatment (interaction tem between parity and treatment *P*=0.67).

## Discussion

The results of this study show a clear effect of using rubber mats in combination with zinc ointment for sows with shoulder lesions compared to antibiotic spray. The effect of treatment was statistical significant for lean sows both when the size of the ulcer on day 14 and day 21 were used as responses. For fat sows, the effect was significant when the size of the lesion on day 21 was used as the response, but not when the size of the lesion on day 14 was used. There was no statistical significant effect of parity on the size of the ulcer on day 14, nor on the effect of treatment. There was an effect of parity on the size of the ulcer on day 21, but this effect was correlated with body condition, and therefore the model including treatment and body condition was preferred. There was a minor difference between herds regarding the size of the ulcer both on day 14 and day 21, but herd did not impact on the effect of treatment on the size of the ulcer. This should not be confused with an effect of the risk of a sow developing a shoulder ulcer. The larger the ulcer was on day 1, the larger it was on day 14 and day 21. This implies how important it is to prevent further development at the first signs of an ulcer. Furthermore, farmers must understand that although an old sow has a higher probability of developing a shoulder ulcer, the young sow with a shoulder ulcer will still benefit from a rubber mat. Mats are therefore recommended as a means to reduce the number of sows that need to be euthanised, culled and weaned too early due to this type of lesion. It is also reasonable to expect that mats could be used preventively for sows at risk.

There is only little documentation of how soft a mat must be before a therapeutic effect can be seen. A single study has found that rubber mat with a foam core had preventive effect against shoulder ulcers in one out of two herds, compared with a solid floor of concrete and solid rubber mats [15]. In the present study, the farmers had no difficulty in keeping the pens clean and dry when using these mats. The mats were fixed to the floor using a special lock system, which held the mats adequately in place and was relatively easy to handle. Therefore, the farmers were generally satisfied with the system.

In Denmark, all use of antibiotic treatment is on prescription only and is monitored in the Vetstat Database [16]. Danish farmers therefore use various non-antibiotic alternatives for wound healing. One such common treatment is zinc ointment, and farmers have reported positive results from this for treatment of shoulder ulcers. In human medicine, zinc ointment is sometimes used prophylactically to protect the skin against moisture and urine, though decompression is considered to be the main therapy for patients with pressure sores [8]. However, there is little documentation on the effect of zinc ointment.

In our study, sows in group A had to be treated due to animal welfare considerations. Although the effect of antibiotic spray treatment on chronic lesions has not been well documented, it should be a reasonable choice for topical treatment. Therefore, it was decided to compare current practice (rubber mats and zinc ointment) with antibiotic spray treatment. It may be open to discussion whether it is at all necessary to use local treatment on shoulder ulcers. It is, nevertheless, a limitation that the study is unable to estimate the effect of rubber mats and zinc treatment separately, although we believe that the observed effect is mainly related to the use of rubber mats. At least the study shows that antibiotics for topical use are inadequate if shoulder lesions are to be prevented from getting worse.

The effect of the treatment on shoulder ulcers was only documented for sows on fully slatted floors which are a very common type of flooring used in intensive pig production world-wide. It is assumed that a fully slatted floor puts more pressure on the shoulder than other types of floors, because the surface area on which the sow lies is smaller. Zubrigg [9] has shown that shoulder ulcers in sows standing on rubber mats healed significantly faster than ulcers in sows standing on steel sheets or a fully slatted floor. It is therefore likely that mats have an effect in pens with other types of flooring. The type of floor must be considered as part of a multi-factorial complex, and several measures need to be taken to prevent sows from developing shoulder ulcers.

Field trials are complicated and expensive to conduct. They usually result in only a limited number of observations being included in the final study, which might reduce the ability to identify important statistical associations. The allocation into treatment groups was performed at random (and matched according to lesion severity represented by grade of ulcer), without taking any covariates into consideration. This randomisation does not guarantee that all covariates will be equally distributed in group M. Therefore, analytical control of potential confounding variables such as parity and body condition was performed. This approach is recommended for use in large field trials (N>100) with relatively homogenous experimental units, as seen in our field trial [17].

The study was primarily conducted during the coldest period of the year. Unfortunately, this made it impossible to estimate any seasonal variation.

## Conclusions

It can be concluded that rubber mats in combination with zinc ointment reduced tissue damage and reduced the area of shoulder ulcers in sows when compared with topical antibiotic treatment alone. The treatment reduced the progression of shoulder lesions when it was used in a pen with a fully slatted floor.

## Competing interests

The authors declare that they have no competing interests.

## Authors’ contributions

MK: The main initiator, performer of the study and author of the manuscript; CSK, PB, LA contributed to designing, and performing the study and revising the manuscript. LA carried out the statistical analyses. All authors have read and approved the manuscript.

## Supplementary Material

Additional file 1 Pharmacy baby zinc ointment ingredients. Click here for file
