# Supplementary material for: Treatment of shoulder ulcers in sows – rubber mats and zinc ointment compared to chlortetracycline spray
Source: Acta Vet Scand. 2013 Feb 15;55(1):12. doi: 10.1186/1751-0147-55-12 (PMC3629990; doi:10.1186/1751-0147-55-12)
Supplement: Additional file 1 — Pharmacy baby zinc ointment ingredients. [file 1751-0147-55-12-S1.pdf]

Pharmacy Baby Zinc Ointment Ingredients

Acqua
Zink Oxide
Petrolatum
Octyldodecanol
Glycerine
Polyglyceryl-4 Diisostearate/Polystearate/ Sebacate
Polyhydroxystearic Acid
Glyceryl Stearate. Sorbitan Isostearate
Zinc Stearate. Magnesium Sulfate
Sodium Gluconate
Fat content: 32%.
